# Clinical Decision Support Tools in the Electronic Medical Record

**DOI:** 10.1016/j.ekir.2023.10.019

**Published:** 2023-10-29

**Authors:** Mackenzie Alexiuk, Heba Elgubtan, Navdeep Tangri

**Affiliations:** 1 Chronic Disease Innovation Centre, Winnipeg, Manitoba, Canada; 2 Community Health Sciences, Max Rady College of Medicine, University of Manitoba, Winnipeg, Manitoba, Canada; 3 Department of Internal Medicine, Max Rady College of Medicine, University of Manitoba, Winnipeg, Manitoba, Canada

**Keywords:** clinical decision support tools, electronic medical records, Kidney Failure Risk Equation, predictive models, risk prediction, Statin Choice Decision Aid

## Abstract

The integration of clinical decision support (CDS) tools into electronic medical record (EMR) systems has become common. Although there are many benefits for both patients and providers from successful integration, barriers exist that prevent consistent and effective use of these tools. Such barriers include tool alert fatigue, lack of interoperability between tools and medical record systems, and poor acceptance of tools by care providers. However, successful integration of CDS tools into EMR systems have been reported; examples of these include the Statin Choice Decision Aid, and the Kidney Failure Risk Equation (KFRE). This article reviews the history of EMR systems and its integration with CDS tools, the barriers preventing successful integration, and the benefits reported from successful integration. This article also provides suggestions and strategies for improving successful integration, making these tools easier to use and more effective for care providers.

EMR-integrated CDS tools have widely proliferated in the past 20 years by leveraging EMRs to provide evidence-based guidance and enhance clinical decision-making. EMR-integrated CDS tools refer to computer-based functionalities that are seamlessly integrated within EMR platforms.^1^ This is accomplished by utilizing patient-specific EMR data in a computerized knowledge base, which provides evidence-based recommendations to the clinician to guide their decision.^1^ These tools have been primarily designed to be used at the point of care, specifically for the clinician to integrate their knowledge with the output of recommendations from algorithms and knowledge-based engines.^2^^,^^3^

The purpose of this review is to identify examples of EMR-integrated CDS tools such as the Statin Choice Decision Aid and KFRE, and provide evidence and guidance on the implementation of future tools in cardiovascular (CV) and kidney disease. Our goal is to highlight the potential benefits of these tools, their role in improving patient outcomes, and the barriers that need to be addressed for successful integration into clinical workflows.

### History of EMRs

The origins of EMRs can be traced back to the development of early computer systems and information technology.^4^ In the 1960s, researchers and hospitals began experimenting with computerized databases as a method of storing patient information.^5^ Notably in 1967, Dr. Lawrence L. Weed, a physician at the University of Vermont developed the Problem-Oriented Medical Information System,^5^^,^^6^ which was one of the first computer-based medical record systems designed to electronically catalog patient data.^5^ The first EMR system was developed in 1972 by the Regenstreif Institute in the United States.^4^^,^^6^ However, the adoption of this EMR system was limited due to the prohibitive cost of implementation and the lack of adequate technological infrastructure across health institutions at the time.^5^^,^^6^

The rising popularity of computers and technological advancements throughout the 1980s and 1990s established the foundation for widespread adoption of EMRs into hospitals.^2^^,^^4^ However, it was not until the 2000s when an organization such as Health Level Seven International emphasized the importance of EMR use for standardization and interoperability.^7^ Moreover, several global organizations established standardized protocols for medical terminology and data exchange, which paved the way for a more efficient integration of EMRs into health care systems throughout the past decade.^7^

During the initial stages, EMRs were primarily used for physician note-taking and billing processes.^4^ EMRs transformed the process of collecting patient information as it began to transition away from paper-based formats.^4^ Digital EMRs provided physicians with platforms equipped with structured templates and standardized data fields, that facilitated more continuity and organization when documenting patient information.^2^^,^^4^^,^^7^ Billing procedures subsequently evolved into more streamlined processes, allowing health care providers to generate electronic claims using data directly from the patient’s EMR.^4^

There are instances when health technology companies have effectively used EMRs for the purpose of clinical research.^8^^,^^9^ These companies utilize their EMR platforms to gather patient data, including aspects such as demographic information, treatment plans, clinical outcomes, and genomic profiles from health care providers.^9^ One notable advantage of this EMR-based approach is its ability to bridge the gap between clinical trials and real-world practice.^9^ EMR-based research has the capacity to facilitate large-scale retrospective studies and the development of machine learning algorithms to explore disease trends and assess the effectiveness of novel therapies and current treatment plans.^9^ Furthermore, research using EMRs can be leveraged in CDS and evaluating risk prediction.^9^

Despite their benefits, the potential for CDS using EMR systems continues to be underutilized. EMRs contain a magnitude of patient data that is often not optimally harnessed for efficient CDS. EMR-integrated CDS tools offer a diverse range of functionalities that aid in clinical decision-making and enhance patient care.^4^^,^^10^ These tools provide clinicians with drug interaction alerts, support diagnosis, guide treatment recommendations based on evidence-based guidelines, and enable proactive disease surveillance.^4^ The benefits of these tools include improved patient safety, enhanced adherence to evidence-based practices, and reduced medical errors.^10^

### Barriers to Successful Integration Of CDS Tools In The EMR

Regardless of the potential benefits that CDS tools can provide clinicians, very few CDS tools have been successfully integrated into EMRs.^10^ There are several reasons an EMR-integrated CDS tool would not be beneficial nor successful.^10^, ^11^, ^12^ These reasons can include the demographics of patients, behavioral patterns of patients and providers, and the effectiveness of a tool outside of CDS tool development.^10^^,^^11^^,^^13^ However, we believe that following 4 elements associated with EMR-integrated CDS tools are the primary barriers to successful implementation:

#### Alert Fatigue

Research has shown that a considerable proportion of alerts generated by EMR-integrated CDS tools are deemed insignificant or inconsequential.^10^^,^^14^, ^15^, ^16^ Furthermore, reports show that clinicians frequently express a disagreement with or develop a lack of trust in the alerts as they surge in volume.^10^^,^^14^^,^^15^^,^^17^ In some circumstances, the magnitude of the alerts results in clinicians potentially being incapable of addressing the alerts in a timely manner.^10^^,^^15^^,^^17^ Dealing with excessive alerts can often result in alert fatigue.^14^ One study recommends that alerts should be stratified into the following 3 tiers based on their purpose: (i) a critical alert, where immediate action is required; (ii) a daily alert, which can be reviewed by staff on a daily basis but does not require urgent attention; and (iii) a noncritical alert, which can be reviewed on a weekly basis.^18^ In addition, alerts regarding medications can vary depending on the specialty and potentially lose their relevance when interpreted out of context.^10^^,^^14^, ^15^, ^16^^,^^18^ For example, an alert regarding duplicate medications may not be applicable in certain specialties, where multiple administration routes of the same drug may be used to enhance the effectiveness of the therapy.^10^^,^^18^

#### Impact of Poor-Quality Evidence Base

The effectiveness of EMR-integrated CDS tools relies upon the quality of the evidence base used to create them.^13^^,^^19^ A poor-quality evidence base for EMR-integrated CDS tools is a critical concern in health care settings.^10^^,^^19^ Inaccurate recommendations may arise from biased or flawed studies, which could potentially lead to inappropriate clinical decisions.^19^^,^^20^ Therefore, if the evidence supporting CDS tools is of low quality, it can result in significant implications for patient care.^19^ Furthermore, if the evidence base lacks generalizability, it can limit the applicability of the CDS tools’ output to diverse patient populations, resulting in the risk of delivering suboptimal care for patients.^19^^,^^20^ EMR-integrated CDS tools depend on centralized data repositories to determine their quality.^19^ Therefore, the quality of data can impact the quality of decision support.^19^ A CDS tool may still be designed for use at the point of care; however, it will be difficult for the tool to be applicable in practice.^19^

#### Lack of Interoperability

The lack of interoperability of EMR-integrated CDS tools is a significant challenge that hinders their ability to smoothly integrate into health care systems.^10^^,^^21^, ^22^, ^23^ Interoperability is defined as the ability of various systems to exchange and utilize data in an efficient and effective manner.^10^ In the context of EMR-integrated CDS tools, interoperability involves the continuous exchange of patient data between EMR systems and the CDS tool.^21^^,^^23^^,^^24^ As a result, clinicians can use the CDS tool in an effective manner to produce recommendations and alerts based on the specific EMR data of the patient.^21^

One of the primary barriers to interoperability is the absence of standardized data formats among various EMR systems.^10^^,^^13^^,^^25^ Health care organizations often utilize a different EMR system that comes with its own technology, data structure, and coding system.^21^^,^^25^^,^^26^ Therefore, the variation in the data structures makes it difficult for CDS tools to integrate seamlessly across the various systems.^13^^,^^24^ This is because data are required to be reformatted to match the EMR system to output meaningful decision support.^10^ The lack of standardized EMR systems poses a challenge to interoperability because it hinders the ability of the clinician to retrieve the necessary patient data from the EMR system to input into the CDS tool, and that can lead to incomplete information or delays in the clinical decision-making process.^21^

#### User Acceptance and Workflow Integration

The success of EMR-integrated CDS tools hinges on their degree of integration into existing clinical workflows. Workflow integration refers to the incorporation of CDS tools into existing clinical processes and ensuring that the tools are capable of fitting into the flow of health care delivery.^16^^,^^27^ Health care professionals are key stakeholders in the integration of CDS tools; therefore, their engagement is imperative to the efficient utilization.^16^^,^^28^^,^^29^ Clinicians are accustomed to their streamlined routines when providing care to their patients.^16^^,^^28^ Consequently, if the CDS tools require additional steps that disrupt the natural flow of care, they could potentially be an additional burden added to the clinician’s practice.^12^^,^^28^ This can result in limited engagement with the CDS tools, which undermines their potential benefits.^16^^,^^23^^,^^28^

The lack of user acceptance, or the resistance to change also contributes to the barrier of workflow integration.^10^^,^^23^ Clinicians may be hesitant to adapt to new CDS tools or change their established procedures, especially if CDS tools are viewed as a disruption to their decision-making methods.^23^ Thus, education and user training are essential to address these concerns while promoting widespread acceptance of EMR-integrated CDS tools within workflows.^10^

### Examples of Successful Integration

#### Statin Choice Decision Aid

Statins have become one of many standard therapies for the management of cholesterol and the prevention of major adverse CV event.^30^ Current guidelines recommend that patients with type 2 diabetes mellitus use statins to mitigate overall CV risk.^31^ However, determining whether to initiate statin treatment demands thorough consideration of individual patient characteristics.^31^, ^32^, ^33^, ^34^, ^35^ The Statin Choice Decision Aid, developed by researchers at the Mayo Clinic in 2007, is an EMR-integrated CDS tool that is designed to aid health care providers in navigating cholesterol management among patients with diabetes.^31^^,^^32^^,^^36^^,^^37^ Through utilizing patient-specific data from the EMR, this decision aid calculates personalized risk assessments by using one of the following 3 risk calculators: (i) ACC/AHA pooled cohort equation for atherosclerotic CV disease, (ii) Framingham risk score, or (iii) Reynolds risk score.^37^ Each risk calculator incorporates factors such as age, gender (as male or female), lipid profile, systolic blood pressure, comorbidities, and lifestyle behaviors to calculate the patient’s baseline 10-year CV risk.^37^ Further, the decision aid automatically catalogs the estimated CV risk into 3 categories: where 10% accounts for patients with a CV risk of <15%, 20% is for patients with an estimated CV risk between 15% and 30%, and 50% risk for patients with an estimated CV risk >30%.^32^^,^^36^^,^^37^ With the option to integrate the decision aid into EMR systems, it can automatically calculate CV risk using real-time patient data, providing patients with accurate risk assessment and a thorough understanding of statin therapy benefits for each patient.^31^, ^32^, ^33^^,^^36^ The integration of the Statin Choice Decision Aid within EMR systems is a notable strength of this CDS tool because it also allows for more streamlined documentation of statin therapy decisions, facilitating enhanced continuity of care and overall communication between health care providers.^31^, ^32^, ^33^, ^34^^,^^36^

#### Kidney Failure Risk Equation (KFRE)

There are a variety of nephrology CDS tools that have been integrated into EMR systems, including for chronic kidney disease (CKD),^38^, ^39^, ^40^ acute kidney injury,^41^ and others.^42^ Web-based tools also exist, including the Decision Aid for Renal Therapy and the QKidney Calculator.^40^ The KFRE, developed in 2011, is one such tool which calculates the risk of developing kidney failure, which requires kidney replacement therapy, in 2 and 5 years in patients living with stages G3–G5 CKD.^43^^,^^44^ In its simplest form, the 4-variable KFRE model incorporates age, sex, estimated glomerular filtration rate, and urinary albumin-to-creatinine ratio; however, an 8-variable model also exists, which incorporates additional metrics of calcium, phosphorus, bicarbonate, and albumin values.^43^^,^^44^ The 4 variables included within the simplified KFRE equation, with the exception of urinary albumin-to-creatinine ratio in some jurisdictions, are routinely collected and accessible in an EMR system.^45^, ^46^, ^47^ Because of the simplicity of the KFRE and accessibility of the variables included in the KFRE, it has previously been implemented into EMR systems to support both clinical practice and research on CDS tool integration.^38^^,^^45^^,^^46^^,^^48^, ^49^, ^50^, ^51^, ^52^, ^53^, ^54^, ^55^, ^56^, ^57^, ^58^, ^59^, ^60^ Via its integration with EMR systems, KFRE can be automatically calculated, improving the identification of patients at risk of developing kidney failure and improving provider workflow.^45^, ^46^, ^47^^,^^49^^,^^52^^,^^53^^,^^55^^,^^57^^,^^60^^,^^61^ Others^45^^,^^47^ have suggested that incorporating automatic prompts into a KFRE CDS tool to encourage providers to order urinary albumin-to-creatinine ratio testing, which is not routinely collected, would improve the accuracy of KFRE and downstream patient outcomes.

Currently, the KFRE is the most widely used prediction model in nephrology practices and in clinical research.^46^^,^^50^^,^^53^^,^^55^, ^56^, ^57^, ^58^, ^59^^,^^62^, ^63^, ^64^, ^65^, ^66^ However, most patients with early-stage CKD and at risk of developing end-stage kidney disease receive care and disease management through their primary care provider.^38^^,^^42^^,^^43^^,^^46^^,^^50^^,^^53^^,^^55^^,^^56^^,^^59^^,^^67^^,^^68^ Unfortunately, these primary-care-managed patients with CKD often receive suboptimal treatment because of their providers’ limited understanding of CKD, its treatment options, and general poor clinical guideline adherence.^10^^,^^38^^,^^42^^,^^50^^,^^51^^,^^68^^,^^69^ The use of KFRE is limited among primary care providers, resulting in patients rarely receiving referrals to nephrology, or being referred much later than is ideal for disease management.^53^^,^^58^^,^^70^, ^71^, ^72^ Like many other CDS tools, there is enormous potential to improve patient outcomes and provider workflows through KFRE integration in primary care settings, as previous research involving this tool has shown promising results.^56^

### Evidence of the Clinical Effectiveness of EMR-Based CDS Tools

Despite barriers, there is a growing body of evidence in favor of incorporating CDS tools into EMR systems. Research exists and more is underway in assessing the incorporation of the KFRE into EMR systems for more accurate and efficient identification of patients at risk for developing kidney failure.

A recent study by researchers at Johns Hopkins University reported positive findings with its incorporation of the KFRE into their Epic EMR system.^53^ Their study participants of faculty and fellow nephrologists highlighted the benefits of automatic calculation to improve their workflow, assist in kidney replacement therapy planning, and summarized possible health outcomes for their patients and nonnephrology care providers.^53^ In addition to displaying both 2-year and 5-year risk of kidney failure, the automatic calculation of KFRE by the EMR system additionally allows for a consistent measure of kidney failure risk over time, and displays patient medications and other kidney events, including renal biopsies and emergency department visits, as displayed in Figure 1.^53^

**Figure 1:**
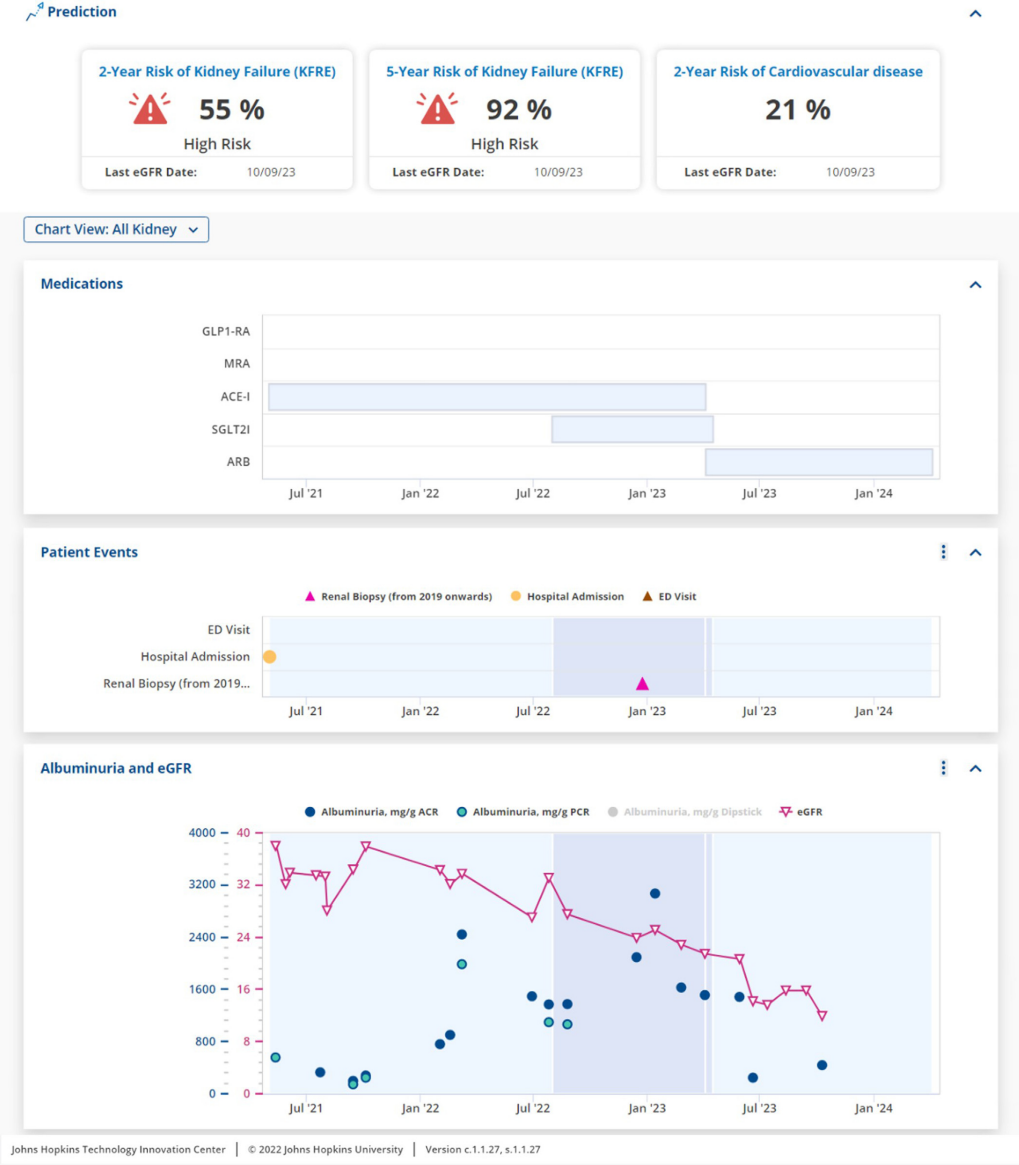

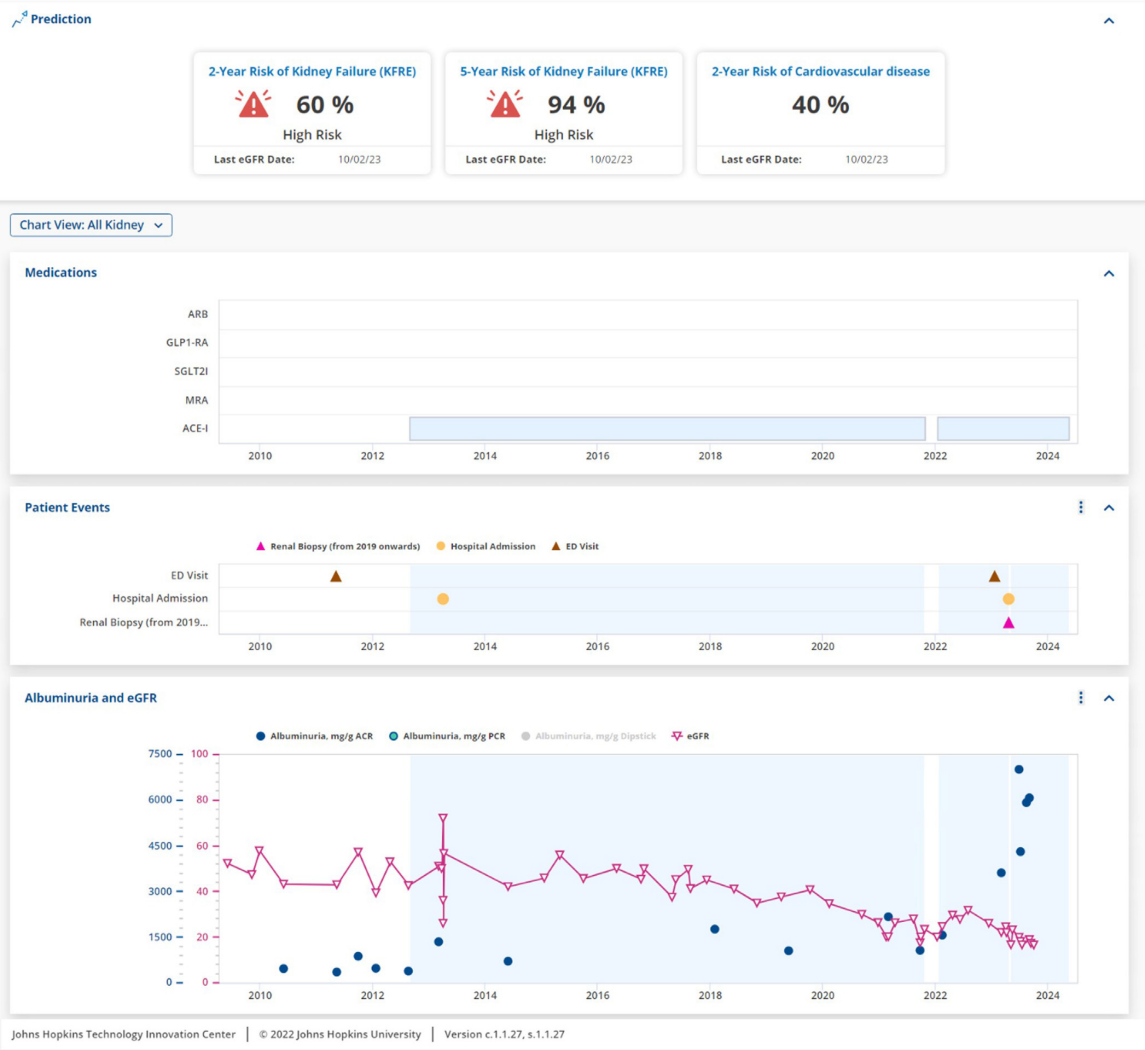
Sample dashboard display of the Kidney Failure Risk Equation integrated into an electronic medical record system. ACEi, angiotensin-converting enzyme inhibitor; ACR, albumin-to-creatinine ratio; ARB, angiotensin receptor blockers; ED, emergency department; eGFR, estimated glomerular filtration rate; GLP1-RA, glucagon-like peptide-1 receptor agonists; MRA, mineralocorticoid receptor antagonists; PCR, protein-to-creatinine ratio; SGLT2i, sodium glucose cotransporter-2 inhibitor. Images courtesy of Johns Hopkins Precision Medicine Center for Kidney Disease.^53^

One study’s use of KFRE to identify those at risk of developing kidney failure found the UK National Institute of Health and Care Excellence 2014 guidelines missed approximately 40% of patients with a >3% risk of end-stage kidney disease for referral to nephrology.^61^ Another study used EMR records to identify necessary referrals to nephrology in primary care and saw an increase from 68 to 94 referrals per month while allowing for more effective triage processes to ensure those who are at greatest risk of end-stage renal disease have access to a nephrologist.^48^ Further, in a study of 201 primary care clinics in Ontario, Canada, the embedding of KFRE into an EMR system allowed for the efficient identification of 361,299 patients.^51^ Of the 361,2999 patients identified, 8194 patients were flagged as being at risk for developing kidney failure, with over 3000 being at moderate or high risk.^51^ Given the challenges associated with identifying individuals at risk for kidney failure, these findings support KFRE-EMR integration because these newly identified patients may not have received care and treatment for their kidneys until failure has progressed.^48^^,^^51^^,^^61^ These findings are also comparable to various other ongoing and completed studies in North America, Asia, the United Kingdom, and Australia.^50^^,^^58^^,^^59^^,^^66^^,^^72^

The use of the Statin Choice Decision Aid in clinical decision making, and disease management also provides substantial evidence-based support from numerous clinical trials.^32^^,^^34^^,^^36^^,^^73^^,^^74^ In a randomized controlled trial of 98 patients assessing the efficacy of the Statin Choice Decision Aid in CDS and treatment adherence, researchers found that the decision aid proved superior compared to pamphlets when informing patients of their CV risk before and after statin use.^32^ Furthermore, the study describes that patients were 6.7 times more likely to understand the degree of CV risk reduction with statin use (odds ratio 6.7; 95% confidence interval 2.2–19.7) following the use of the decision aid.^32^ For patients not receiving statin therapy at baseline, it was reported that 30% of patients in the decision aid group decided to begin statin therapy immediately following their visit using the decision aid with their provider.^32^ Another study found that patients that received the decision aid were more likely to accurately perceive their underlying risk of CV events without taking a statin.^36^ Furthermore, a cluster randomized trial of patients with type 2 diabetes mellitus found that patients who received the decision aid during their visit had a more accurate perception of their 10-year CV risk with and without statin use.^73^ However, there were no significant changes in treatment adherence after 3 months.^73^ Overall, the use of the decision aid significantly reduced decisional conflict among patients and resulted in more starts of stating therapy among patients with a 10-year CV risk >15%.^32^^,^^33^^,^^36^^,^^73^ Acknowledging the challenges associated with CV risk reduction, these findings are consistent with the argument to support the integration of the Statin Choice Decision Aid with EMR, because patients were able to identify their level of CV risk and improve their risk reduction with statin therapies.^32^, ^33^, ^34^^,^^36^^,^^73^, ^74^, ^75^

Beyond KFRE and the Statin Choice Decision Aid, research assessing other CDS tools have been performed. The integration of the Acute Kidney Injury CDS tool into EMRs found statistically significant evidence that the use of an acute kidney injury CDS alert tool resulted in higher risk of death compared to the control arm.^76^ However, the authors note that acute kidney injury alerts may have resulted in providers being distracted from providing wholistic care or would encourage providers to practice in a manner they would not otherwise for fear of legal consequences.^76^ In research using EMR-integrated CDS tools to support prescribing practices, a study found statistically significant but marginal improvements in patient outcomes in those with atherosclerotic CV disease.^77^ Another study focused on CDS tools to support prescribing practices for conditions susceptible to antibiotic resistance, an EMR-integrated CDS alert resulted in providers changing their prescription habits 60% of the time, which resulted in decreased length of hospital stays for some study conditions, but not all.^78^ Finally, in a study which used EMR-integrated CDS tools to encourage providers to use contextualizing care techniques, the frequency of this practice increased; however, patient outcomes showed no significant change.^79^

Among these studies, varying degrees of success are reported; however, positive outcomes have been noted in these works except one. It is important to consider that the effectiveness of EMR-CDS integration may depend on the disease, its progression, its available treatment options, and potential complications when comorbidity is present.^76^, ^77^, ^78^, ^79^

### Guidance for CDS Integration

Regardless of complications surrounding EMR-integrated CDS tools, providers continue to display desire for development, improvement, and access to these tools.^68^ Integration of CDS tools into EMR systems shows promise to provide better care for patients, while simultaneously streamlining clinician practice. However, there is still much work to do to improve these systems and address barriers to ensure provider usage. In addition to the recommendations of automatic calculation, appropriate alerts for KFRE evaluation, and prompts to include urinary albumin-to-creatinine ratio in laboratory testing, we propose the following 5 strategies for the development of better EMR-integrated CDS tools.

#### Integrate Multiple Tools

Beyond the KFRE and Statin Choice Decision Aid, multiple CDS tools must be integrated and accessible in EMR systems. This is especially pertinent when risk factors for one disease may contribute to risk factors for another, as reflected in the relationship with diabetes and CKD. Integration of multiple tools also allows for more comprehensive care from all providers and encourages providers to follow clinical guidelines regarding medication regimens, referrals, and other treatment planning.

#### Easy Integration Into Clinical Charts and Notes

A current barrier to using CDS tools is the requirement for manual data entry into calculation tools external to the EMR system.^45^^,^^47^^,^^52^^,^^55^^,^^57^^,^^61^ If providers must manually transfer output results into their clinical charts and notes, this could arise as another barrier to using CDS tools. Further, if the results received from a CDS tool are complex, the time necessary for transferring the data into clinical charts or notes will likely prevent providers from using these tools further. It is essential that these tools provide their test results efficiently and effectively for the provider and their unique workflow.

#### Portability Between EMR Systems

There are many different EMR systems in use globally, which all have different interface and operating systems for different health care-related needs. As a result, clinics and hospitals may choose one of several EMR systems that best aligns with their financial means and clinical practice. Therefore, CDS tools must be available for all EMR systems to ensure that their patients have access to the most comprehensive care regardless of the system a practice uses. Further, because the EMR system used varies between clinics and hospitals, the CDS results should be viewable on as many EMR platforms as possible to ensure patient care is continuous between settings.

#### Embedding of Relevant Clinical Practice Guidelines

As mentioned in the first proposed future direction, CDS tools allow for easy access to current and accurate clinical guidelines. Considering that clinical guidelines change between regions of the world because of different standard practices and different populations, it is critical that clinical guidelines relevant to each region can be embedded accordingly. Further, as best clinical practices change with advances in medical research, the embedded clinical guidelines must adapt in tandem. The ability to provide clinicians with their relevant and up-to-date clinical guidelines will allow patients to receive the highest levels of care.

#### Application of Large Language Models

Finally, large language models and artificial intelligence should be applied to CDS tools to develop accessible patient-facing materials. Given that health care organizations are advancing toward patient-centered systems where patients are partnered in their own care, sharing information in ways that are accessible to the general population is critical. Large language models and artificial intelligence tools not only assist with calculations for individual patient risk, but also can translate medical jargon into patient facing materials such as websites, pamphlets, and more to help inform patients about their health and their risks for disease.

In summary, CDS can improve both patient care and the provider experience when it is evidence-based, integrated into workflow, and provides clinical utility. Advances in EMRs, modeling methods and large language models will lead to more useful decision support for nephrologists and patients with CKD.

## Disclosure

All the authors declared no competing interests.
